# Supplementary figures and images for: AA-amyloidosis in cats (Felis catus) housed in shelters
Source: PLoS One. 2023 Mar 29;18(3):e0281822. doi: 10.1371/journal.pone.0281822 (PMC10057811; doi:10.1371/journal.pone.0281822)

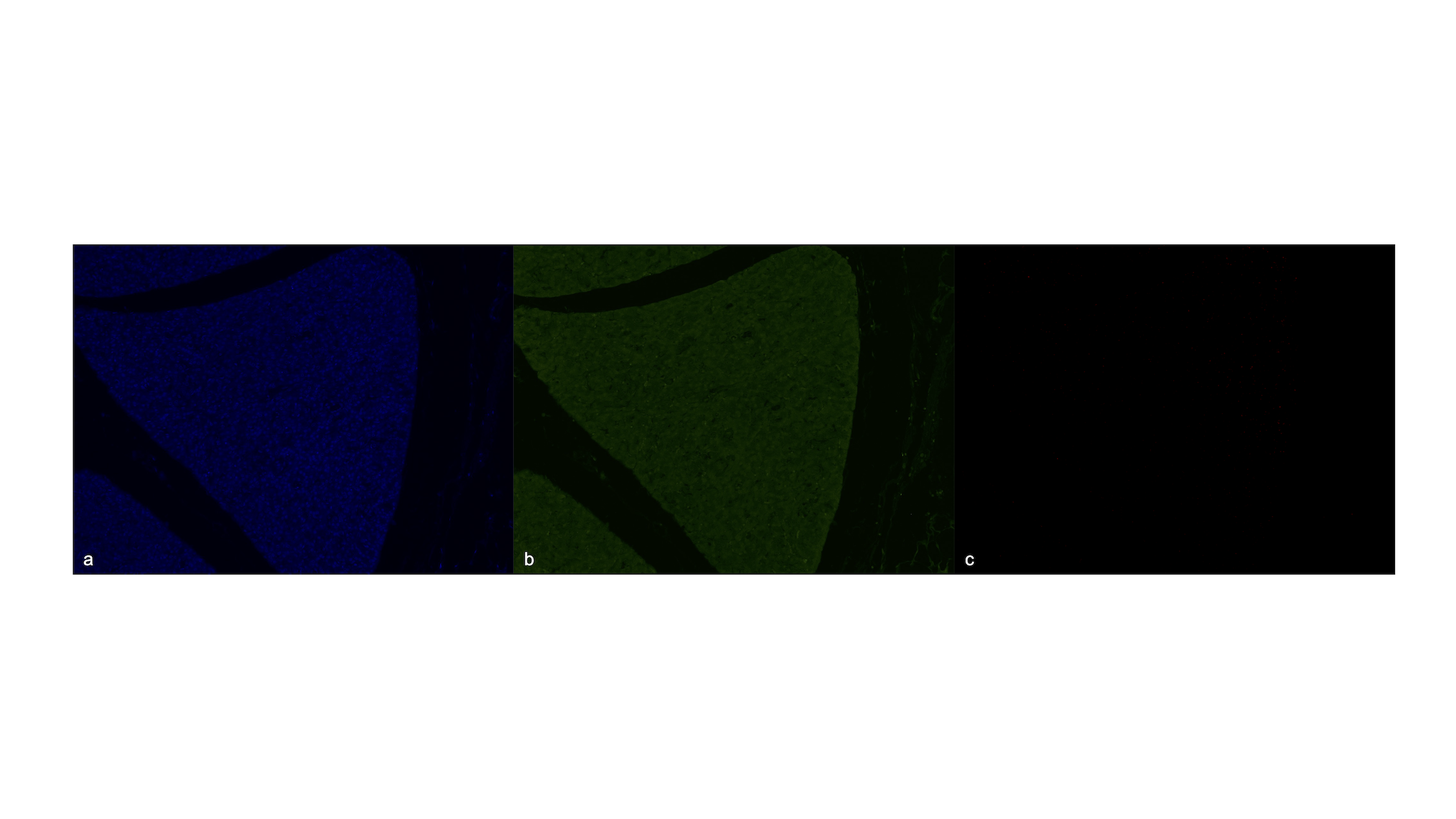

Supplement: S1 Fig — Nuclei are blue with DAPI (a), no amyloid is identified with Thioflavine S (b) or anti-SAA (c). (TIFF) [file pone.0281822.s001.tiff]

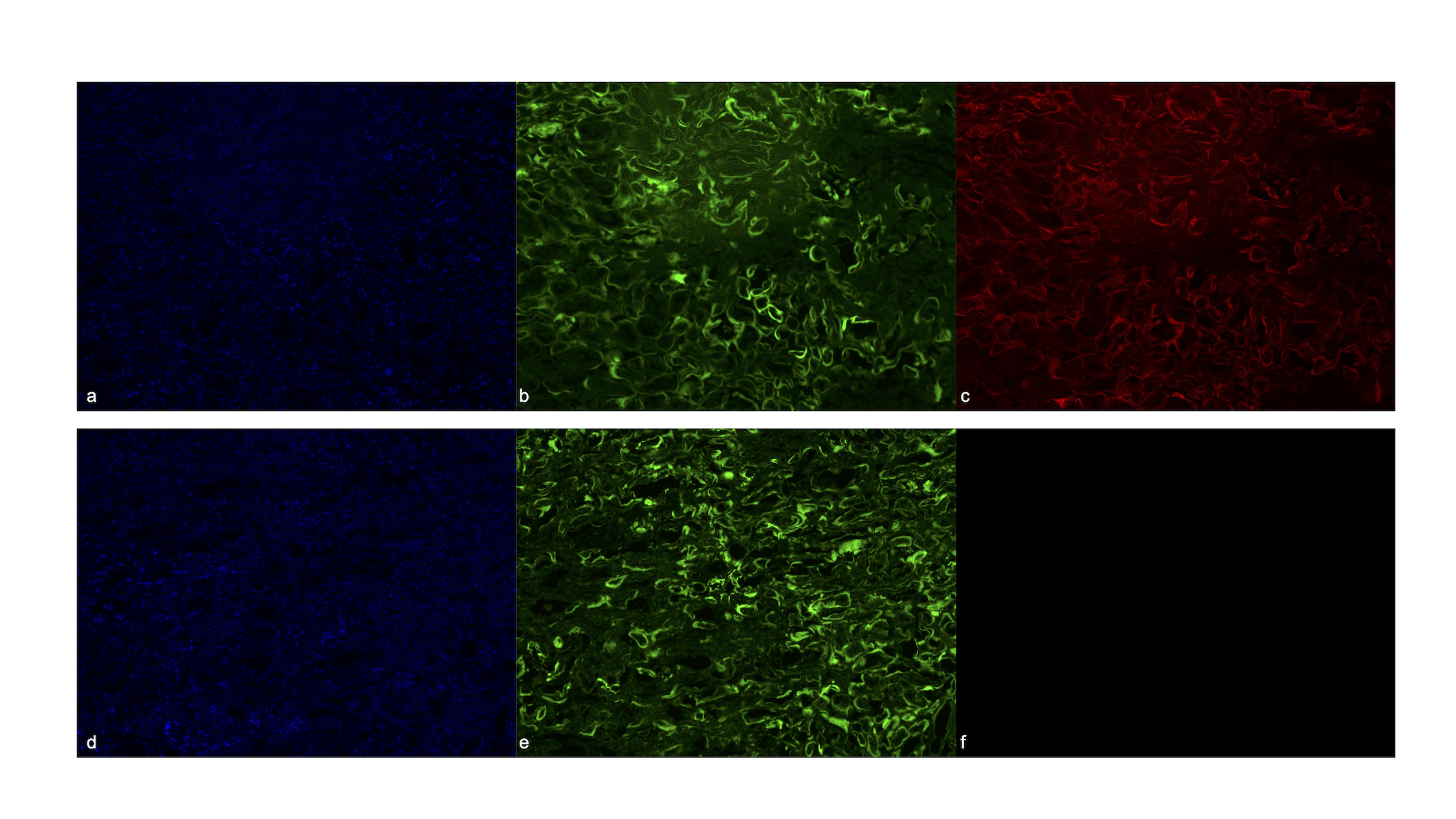

Supplement: S2 Fig — Nuclei are blue with DAPI(a,d; in blue), amyloid is identified with Thioflavine S (b,e; in green) and anti-SAA (c, in red). No signal was detected when the secondary antibody Alexa FluorTM546 was used without anti-SAA (f). (TIFF) [file pone.0281822.s002.tiff]

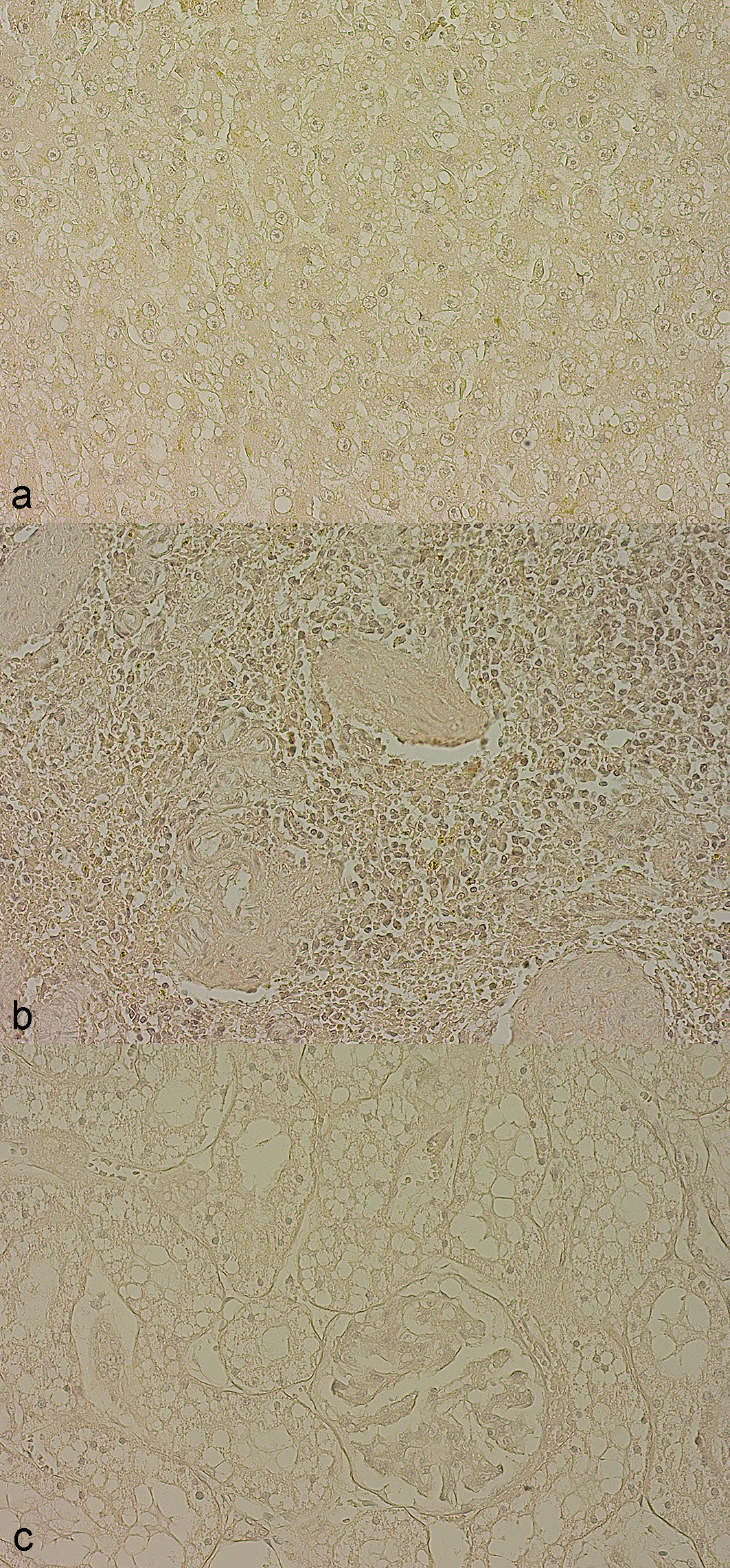

Supplement: S3 Fig — No interstitial red material is detected by Congo red stain. (TIF) [file pone.0281822.s003.tif]

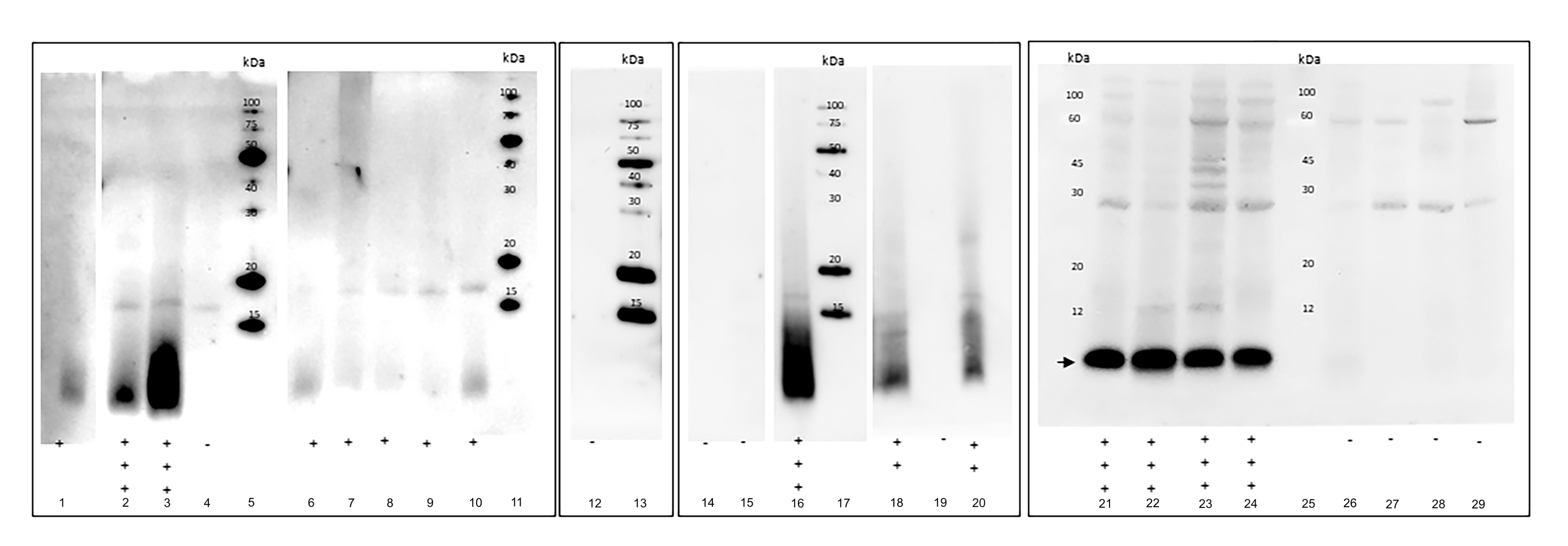

Supplement: S4 Fig — The intensity levels in positive western blot are marked as negative (-), mild (+), moderate (++) and severe (+++). Lanes 5, 11, 13, 17, 25: molecular weight markers; lanes 1–3, 6–10, 16, 18, 20, 21–24: positive bile samples. Lanes 4, 12, 14, 15, 19, 26–29: negative bile samples. (TIF) [file pone.0281822.s004.tif]

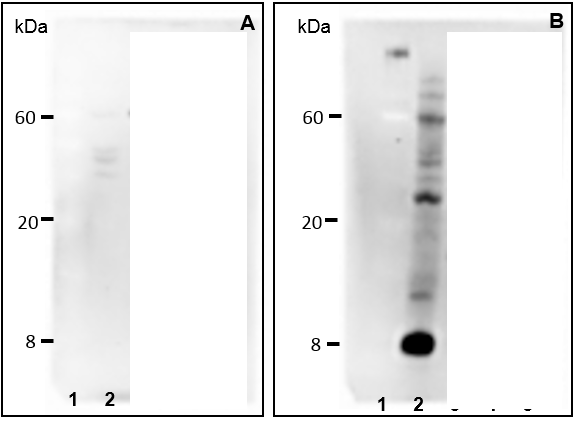

Supplement: S5 Fig — Panel A, with secondary antibody alone. Panel B, with both primary and secondary antibodies. Lane 1: pre-stained molecular markers; lane 2: bile sample with amyloid fragments. (TIF) [file pone.0281822.s005.tif]
