# Supplementary material for: AA-amyloidosis in cats (Felis catus) housed in shelters
Source: PLoS One. 2023 Mar 29;18(3):e0281822. doi: 10.1371/journal.pone.0281822 (PMC10057811; doi:10.1371/journal.pone.0281822)
Supplement: S1 Model — (DOCX) [file pone.0281822.s006.docx]

**S1 Model**. Logistic regression model for AA-amyloidosis status (presence vs absence).

|  | OR | Lower bound | Upper bound | P-value |
| --- | --- | --- | --- | --- |
| Age | 0.963 | 0.775 | 1.197 | 0.735 |
| Duration of stay | 1.014 | 0.995 | 1.034 | 0.108 |
| Shelter A | 1.602 | 0.494 | 5.193 | 0.432 |
| Shelter B | 2.604 | 0.784 | 8.654 | 0.118 |
| Shelter C* |  |  |  | 0.295 |

*Shelter C is the reference level. OR, odd ratio.
