# Supplementary material for: AA-amyloidosis in cats (Felis catus) housed in shelters
Source: PLoS One. 2023 Mar 29;18(3):e0281822. doi: 10.1371/journal.pone.0281822 (PMC10057811; doi:10.1371/journal.pone.0281822)
Supplement: S1 Table — (DOCX) [file pone.0281822.s008.docx]

**S1 Table**. Data of all the included cats: n° of cat, shelter, gender, age at death (years), duration of stay in the shelter (months), presence of amyloidosis in any of the three investigated organs, presence of hepatic amyloidosis, presence of splenic amyloidosis, presence of renal amyloidosis, histological amyloid liver score, histological amyloid splenic score, histological amyloid kidney score, amyloidosis score, results of PCR for FIV, results of PCR for FeLV, results of PCR for FCoV, other chronic concurrent diseases, cause of death, western blot result.

| **N** | **Shelter** | **Gender** | **Age (years)** | **Duration of stay (months)** | **Amyloidosis in any organ (Y/N)** | **Hepatic amyloidosis (Y/N)** | **Splenic amyloidosis (Y/N)** | **Renal amyloidosis (Y/N)** | **Histological amyloid liver score** | **Histological amyloid spleen score** | **Histological amyloid kidney score** | **Amyloidosis score** | **PCR FIV (Y/N)** | **PCR FeLV (Y/N)** | **PCR FCoV (Y/N)** | **Other concurrent diseases** | **Cause of death** | Western blot result |
| --- | --- | --- | --- | --- | --- | --- | --- | --- | --- | --- | --- | --- | --- | --- | --- | --- | --- | --- |
| 1 | A | M | 2 | 7 | N | N | N | N | 0 | 0 | 0 | 0 | N | N | N | Unknown | Seizures | N |
| 2 | A | M | 2 | 8 | N | N | N | N | 0 | 0 | 0 | 0 | N | N | N | Unknown | Feline infectious peritonitis | NA |
| 3 | A | M | 4 | 16 | N | N | N | N | 0 | 0 | 0 | 0 | N | N | N | Unknown | Feline infectious peritonitis | NA |
| 4 | A | M | 4 | 8 | N | N | N | N | 0 | 0 | 0 | 0 | N | N | N | Unknown | Seizures | N |
| 5 | A | M | 4 | 7 | N | N | N | N | 0 | 0 | 0 | 0 | N | N | N | Unknown | Feline infectious peritonitis | NA |
| 6 | A | M | 4 | 12 | N | N | N | N | 0 | 0 | 0 | 0 | N | Y | N | Unknown | Unknown | N |
| 7 | A | M | 4 | 8 | N | N | N | N | 0 | 0 | 0 | 0 | N | N | N | Unknown | Euthanasia | N |
| 8 | A | F | 6 | 15 | N | N | N | N | 0 | 0 | 0 | 0 | N | N | N | Unknown | Severe diarrhea, Inflammatory bowel disease | NA |
| 9 | A | M | 6 | 12 | N | N | N | N | 0 | 0 | 0 | 0 | N | N | N | Unknown | Unknown | NA |
| 10 | A | F | 10 | 9 | N | N | N | N | 0 | 0 | 0 | 0 | N | N | N | Unknown | Kidney failure | NA |
| 11 | A | M | 2 | 2 | N | N | N | N | 0 | 0 | 0 | 0 | NA | NA | NA | Unknown | Unknown | NA |
| 12 | A | F | 4 | 26 | N | N | N | N | 0 | 0 | 0 | 0 | N | N | N | Unknown | Dyspnea/pneumonia | NA |
| 13 | A | M | 5 | 6 | Y | Y | N | N | 1 | 0 | 0 | 1 | Y | N | N | Unknown | Anemia | N |
| 14 | A | F | 6 | 23 | Y | N | Y | N | 0 | 1 | 0 | 1 | N | N | N | Unknown | Dyspnea/pneumonia | NA |
| 15 | A | M | 6 | 68 | Y | Y | Y | Y | 2 | 2 | 2 | 6 | N | Y | N | Unknown | Dyspnea/pneumonia | Y, severe |
| 16 | A | M | 3 | 29 | Y | Y | Y | Y | 2 | 2 | 2 | 6 | N | Y | N | Unknown | Severe diarrhea, Inflammatory bowel disease | NA |
| 17 | A | F | 6 | 66 | Y | N | Y | Y | 0 | 2 | 2 | 4 | N | N | N | Unknown | Severe diarrhea, Inflammatory bowel disease | Y, severe |
| 18 | A | F | 8 | 93 | Y | Y | Y | N | 1 | 1 | 0 | 2 | N | N | N | Unknown | Dyspnea/pneumonia | NA |
| 19 | A | M | 5 | 9 | Y | Y | Y | Y | 1 | 2 | 1 | 4 | N | Y | N | Unknown | Severe diarrhea, Inflammatory bowel disease | Y, severe |
| 20 | A | F | 8 | 27 | Y | Y | Y | Y | 1 | 2 | 2 | 5 | N | N | N | Unknown | Kidney failure | NA |
| 21 | A | F | 6 | 61 | Y | Y | Y | Y | 1 | 2 | 2 | 5 | N | N | N | Unknown | Kidney failure | NA |
| 22 | A | F | 2 | 5 | Y | Y | Y | Y | 2 | 2 | 2 | 6 | N | Y | N | Unknown | Euthanasia | NA |
| 23 | A | F | 5 | 27 | Y | Y | Y | Y | 2 | 2 | 2 | 6 | N | N | N | Unknown | Unknown | NA |
| 24 | A | F | 4 | 20 | Y | N | Y | N | 0 | 2 | 0 | 2 | N | N | N | Unknown | Severe diarrhea, Inflammatory bowel disease | NA |
| 25 | A | M | 5 | 12 | Y | Y | Y | Y | 1 | 2 | 1 | 4 | N | N | N | Unknown | Severe diarrhea, Inflammatory bowel disease | NA |
| 26 | A | F | 2 | 5 | Y | N | Y | Y | 0 | 2 | 1 | 3 | N | N | N | Unknown | Severe diarrhea, Inflammatory bowel disease | NA |
| 27 | A | F | 5 | 34 | Y | N | Y | N | 0 | 2 | 0 | 2 | NA | NA | NA | Unknown | Unknown | NA |
| 28 | A | F | 5 | 46 | Y | Y | Y | Y | 1 | 2 | 2 | 5 | N | N | N | Unknown | Kidney failure | NA |
| 29 | B | M | 10 | 72 | N | N | N | N | 0 | 0 | 0 | 0 | N | Y | N | Unknown | Unknown | NA |
| 30 | B | F | 6 | 12 | N | N | N | N | 0 | 0 | 0 | 0 | N | Y | Y | Unknown | Feline infectious peritonitis | N |
| 31 | B | M | 1 | 9 | N | N | N | N | 0 | 0 | 0 | 0 | N | N | N | Unknown | Feline infectious peritonitis | NA |
| 32 | B | F | 10 | 96 | N | N | N | N | 0 | 0 | 0 | 0 | NA | NA | NA | Unknown | Unknown | NA |
| 33 | B | M | 7 | 6 | N | N | N | N | 0 | 0 | 0 | 0 | NA | NA | NA | Unknown | Unknown | NA |
| 34 | B | M | 6 | 36 | N | N | N | N | 0 | 0 | 0 | 0 | NA | NA | NA | Unknown | Unknown | NA |
| 35 | B | M | 3 | 8 | N | N | N | N | 0 | 0 | 0 | 0 | N | N | N | Unknown | Euthanasia | NA |
| 36 | B | M | 5 | 18 | Y | Y | Y | Y | 1 | 2 | 1 | 4 | N | N | N | Unknown | Severe diarrhea, Inflammatory bowel disease | Y, moderate |
| 37 | B | M | 5 | 2 | Y | N | N | Y | 0 | 0 | 1 | 1 | N | Y | N | Unknown | Euthanasia | NA |
| 38 | B | M | 5 | 40 | Y | Y | Y | Y | 1 | 2 | 2 | 5 | N | N | N | Unknown | Euthanasia | NA |
| 39 | B | M | 5 | 48 | Y | Y | Y | Y | 1 | 2 | 2 | 5 | N | N | N | Unknown | Euthanasia | Y, severe |
| 40 | B | M | 5 | 48 | Y | Y | Y | Y | 2 | 2 | 1 | 5 | N | Y | N | Unknown | Euthanasia | Y, moderate |
| 41 | B | F | 10 | 72 | Y | Y | Y | Y | 1 | 1 | 2 | 4 | N | Y | N | Unknown | Unknown | NA |
| 42 | B | F | 5 | 24 | Y | Y | Y | Y | 1 | 2 | 1 | 4 | N | Y | N | Unknown | Euthanasia | NA |
| 43 | B | F | 5 | 12 | Y | Y | Y | Y | 1 | 2 | 2 | 5 | N | Y | N | Unknown | Unknown | NA |
| 44 | B | F | 5 | 29 | Y | Y | Y | Y | 2 | 2 | 2 | 6 | N | Y | N | Unknown | Feline infectious peritonitis | NA |
| 45 | B | M | 12 | 102 | Y | Y | Y | Y | 2 | 2 | 2 | 6 | N | N | N | Unknown | Unknown | NA |
| 46 | B | M | 9 | 54 | Y | Y | Y | Y | 2 | 1 | 1 | 4 | N | N | N | Unknown | Unknown | NA |
| 47 | B | F | 7 | 36 | Y | N | Y | N | 0 | 2 | 0 | 2 | N | N | N | Unknown | Euthanasia | NA |
| 48 | B | F | 7 | 32 | Y | Y | Y | N | 1 | 2 | 0 | 3 | N | N | N | Unknown | Unknown | NA |
| 49 | B | F | 15 | 144 | Y | Y | Y | Y | 1 | 2 | 2 | 5 | N | N | N | Unknown | Euthanasia | NA |
| 50 | B | M | 6 | 17 | Y | N | Y | Y | 0 | 1 | 2 | 3 | N | Y | N | Unknown | Euthanasia | NA |
| 51 | B | M | 12 | 120 | Y | Y | Y | Y | 1 | 1 | 1 | 3 | N | N | N | Unknown | Euthanasia | NA |
| 52 | B | M | 9 | 72 | Y | Y | Y | Y | 1 | 1 | 2 | 4 | NA | NA | NA | Unknown | Unknown | NA |
| 53 | B | F | 7 | 46 | Y | Y | Y | Y | 2 | 2 | 1 | 5 | NA | NA | NA | Unknown | Unknown | NA |
| 54 | B | M | 12 | 120 | Y | Y | Y | Y | 2 | 2 | 2 | 6 | N | N | Y | Unknown | Unknown | NA |
| 55 | C | F | 8 | 87 | N | N | N | N | 0 | 0 | 0 | 0 | N | N | N | Polycystic kidney disease | Kidney failure | Y, mild |
| 56 | C | M | 7 | 43 | N | N | N | N | 0 | 0 | 0 | 0 | NA | NA | NA | Gingivostomatitis | Kidney failure | Y, mild |
| 57 | C | M | 6 | 7 | N | N | N | N | 0 | 0 | 0 | 0 | N | Y | N | Intestinal lymphoma | Lymphoma | Y, mild |
| 58 | C | F | 10 | 38 | N | N | N | N | 0 | 0 | 0 | 0 | N | N | N | Bone marrow aplasia, epilepsy | Seizures | Y, mild |
| 59 | C | F | 2 | 18 | N | N | N | N | 0 | 0 | 0 | 0 | N | Y | N | Splenic lymphoma, cystitis | Lymphoma | Y, mild |
| 60 | C | F | 17 | 204 | N | N | N | N | 0 | 0 | 0 | 0 | NA | NA | NA | / | Kidney failure | N |
| 61 | C | M | 11 | 8 | N | N | N | N | 0 | 0 | 0 | 0 | N | N | N | Enteropathy | Lymphoma | N |
| 62 | C | M | 1 | 9 | N | N | N | N | 0 | 0 | 0 | 0 | NA | NA | NA | Feline infectious peritonitis | Feline infectious peritonitis | NA |
| 63 | C | M | 5 | 15 | N | N | N | N | 0 | 0 | 0 | 0 | N | Y | N | Feline infectious peritonitis | Feline infectious peritonitis | NA |
| 64 | C | F | 3 | 1 | N | N | N | N | 0 | 0 | 0 | 0 | NA | NA | NA | Unknown | Unknown | NA |
| 65 | C | M | 14 | 130 | N | N | N | N | 0 | 0 | 0 | 0 | NA | NA | NA | Unknown | Dyspnea/pneumonia | NA |
| 66 | C | F | 4 | 23 | N | N | N | N | 0 | 0 | 0 | 0 | N | N | N | Bone marrow aplasia, toxoplasmosis, hemotropic mycoplasma | Bone marrow aplasia | NA |
| 67 | C | F | 7 | 32 | Y | Y | Y | Y | 1 | 2 | 1 | 4 | N | N | N | Toxoplasmosis, enteropathy, , respiratory tract disease (pneumonia) | Hemoabdomen due to hepatic rupture | Y, severe |
| 68 | C | M | 13 | 69 | Y | N | Y | Y | 0 | 2 | 1 | 3 | N | N | N | / | Kidney failure | Y, mild |
| 69 | C | M | 4 | 30 | Y | Y | Y | Y | 2 | 2 | 2 | 6 | N | N | N | Gingivostomatitis, enteropathy | Kidney failure | NA |
| 70 | C | M | 8 | 87 | Y | Y | Y | Y | 1 | 1 | 1 | 3 | N | N | N | Gingivostomatitis | Kidney failure | Y, severe |
| 71 | C | F | 3 | 40 | Y | Y | Y | Y | 2 | 1 | 2 | 5 | NA | NA | NA | Respiratory tract disease (rhinitis), enteropathy | Hemoabdomen due to hepatic rupture | NA |
| 72 | C | F | 13 | 7 | Y | Y | Y | Y | 1 | 1 | 1 | 3 | Y | Y | N | Ccular carcinoma | Unknown | Y, mild |
| 73 | C | M | 6 | 14 | Y | N | Y | N | 0 | 2 | 0 | 2 | Y | Y | N | Gingivostomatitis | Anemia | N |
| 74 | C | M | 8 | 80 | Y | Y | Y | Y | 1 | 2 | 1 | 4 | N | N | N | Enteropathy | Kidney failure | NA |
| 75 | C | F | 8 | 84 | Y | Y | Y | Y | 1 | 2 | 2 | 5 | N | Y | N | Recurrent cutaneous abscess | Kidney failure | NA |
| 76 | C | F | 2 | 13 | Y | Y | Y | Y | 1 | 2 | 2 | 5 | N | N | N | Respiratory tract disease (pneumonia) | Dyspnea/pneumonia | NA |
| 77 | C | F | 12 | 120 | Y | Y | Y | Y | 2 | 2 | 2 | 6 | N | N | N | Gingivostomatis, | Kidney failure | NA |
| 78 | C | M | 4 | 3 | Y | Y | Y | Y | 1 | 2 | 1 | 4 | NA | NA | NA | Unknown | Unknown | NA |
| 79 | C | M | 9 | 99 | Y | Y | Y | Y | 2 | 2 | 2 | 6 | N | N | N | Cystitis | Kidney failure | NA |

Y, positive result; N, negative result; NA, not available; organs histological scores are classified as follow: 0 negative, 1 mildly positive, 2 moderately/severely positive; amyloidosis score is created by adding the histological score of each organ.
