# Supplementary material for: AA-amyloidosis in cats (Felis catus) housed in shelters
Source: PLoS One. 2023 Mar 29;18(3):e0281822. doi: 10.1371/journal.pone.0281822 (PMC10057811; doi:10.1371/journal.pone.0281822)
Supplement: S2 Table — (DOCX) [file pone.0281822.s009.docx]

**S2 Table**. Distribution of the AA-amyloidosis additive score in cats.

| Additive histological score | N (%) |
| --- | --- |
| 0 | 31 (39.2) |
| 1 | 3 (3.8) |
| 2 | 5 (6.3) |
| 3 | 7 (8.9) |
| 4 | 11 (13.9) |
| 5 | 12 (15.2) |
| 6 | 10 (12.7) |
| Total | 79 (100) |

N = number; % = percentage
